# When expectancies collide: Action dynamics reveal the interaction between stimulus plausibility and congruency

**DOI:** 10.3758/s13423-016-1033-6

**Published:** 2016-05-19

**Authors:** Moreno I. Coco, Nicholas D. Duran

**Affiliations:** 1 School of Philosophy, Psychology and Language Sciences, University of Edinburgh, 3 Charles Street, Edinburgh, EH8 9AD UK; 2 School of Social and Behavioral Sciences, Arizona State University, Glendale, AZ 85036 USA

**Keywords:** Event plausibility, Contextual congruency, Cross-modal processing, Verification task, Mouse-tracking

## Abstract

The cognitive architecture routinely relies on expectancy mechanisms to process the plausibility of stimuli and establish their sequential congruency. In two computer mouse-tracking experiments, we use a cross-modal verification task to uncover the interaction between plausibility and congruency by examining their temporal signatures of activation competition as expressed in a computer- mouse movement decision response. In this task, participants verified the content congruency of sentence and scene pairs that varied in plausibility. The order of presentation (sentence-scene, scene-sentence) was varied between participants to uncover any differential processing. Our results show that implausible but congruent stimuli triggered less accurate and slower responses than implausible and incongruent stimuli, and were associated with more complex angular mouse trajectories independent of the order of presentation. This study provides novel evidence of a disassociation between the temporal signatures of plausibility and congruency detection on decision responses.

## Introduction

When experiencing events, our cognitive system routinely makes use of expectations to anticipate upcoming information and to guide action (e.g., Rao & Ballard, 1999; Friston, 2010; Wacongne et al., 2012). The violation of expectations, and their relation to stimulus plausibility, has been explored in areas as diverse as language comprehension to visual scene perception (e.g., Kutas & Hillyard, 1980; Van Berkum et al., 1999; Henderson et al., 1999; Ganis & Kutas, 2003; Hagoort et al., 2004; Mudrik et al., 2010; Võ & Wolfe, 2013; Coco et al., 2014). An open challenge remains to understand how the cognitive system utilizes expectancy mechanisms to synchronously hold information across multiple points in time and integrate it to produce action responses (Bar 2007). The growing attention towards this challenge can be traced to current proposals in the cognitive sciences that aim to bridge low-level perceptual processes, high-level expectancy mechanisms, and motor control within the same predictive processing framework (e.g., Clark, 2013; Pickering & Clark, 2014). Lupyan and Clark (2015), for example, suggest that perception is an inferential process, whereby prior beliefs are combined with incoming sensory data to optimize in-the-moment processing and to improve future predictions. In the current study, we draw from this framework to explore how different types of expectations interactively mediate comprehension, and how these comprehension processes can be captured over time through a fine-grained analysis of response behavior.

Previous research on expectancy mechanisms has largely employed electrophysiology (EEG) measures. A common finding in this research is that a mismatch between predictions and incoming linguistic or non-linguistic stimuli trigger negative shifts in event-related brain potentials (e.g., Ganis & Kutas, 2003; Kutas & Hillyard, 1980; DeLong et al, 2005; Mudrik et al., 2010). Such shifts are typically interpreted as evidence that a prediction had occurred and that prior knowledge has been updated. Moreover, the magnitude of these negative shifts is modulated by the context in which unexpected stimuli is placed (e.g., a sentential context, Marslen-Wilson & Tyler, 1980; Kutas, 1993), as well as by additional information preceding it, such as a narrative of visual scenes (Sitnikova et al. 2008; Cohn et al. 2012).

Although EEG studies have provided invaluable insights into contextual effects and processing costs at the moment unexpected stimuli is encountered, there may also be additional costs that persist after the initial encounter. This is particularly true when expectations must be maintained in memory to perform an explicit decision response, such as in verification tasks where participants are asked to assess the congruency of a pair of sequentially presented stimuli (i.e., whether a sentence and a picture convey the same message or not; e.g., Clark & Chase, 1972; Carpenter & Just, 1975). We hypothesize, per a predictive processing framework, that when incongruent stimuli are encountered, activation elicited from the initial stimulus will compete with bottom-up sensory activation from the second stimulus, taking time to resolve as the error signal is adjusted. Moreover, we expect multiple sources of expectancy to contribute to the prediction activation strength, i.e., not only the congruence between consecutive stimuli, but also their plausibility (i.e., whether the stimuli being conveyed depicts something plausible or implausible). The interaction between these sources has been only marginally investigated and there have been no studies, as far as we are aware, that have examined how the costs driven by congruency and plausibility activate, compete, and are resolved throughout an extended decision response.

**Fig. 1:**
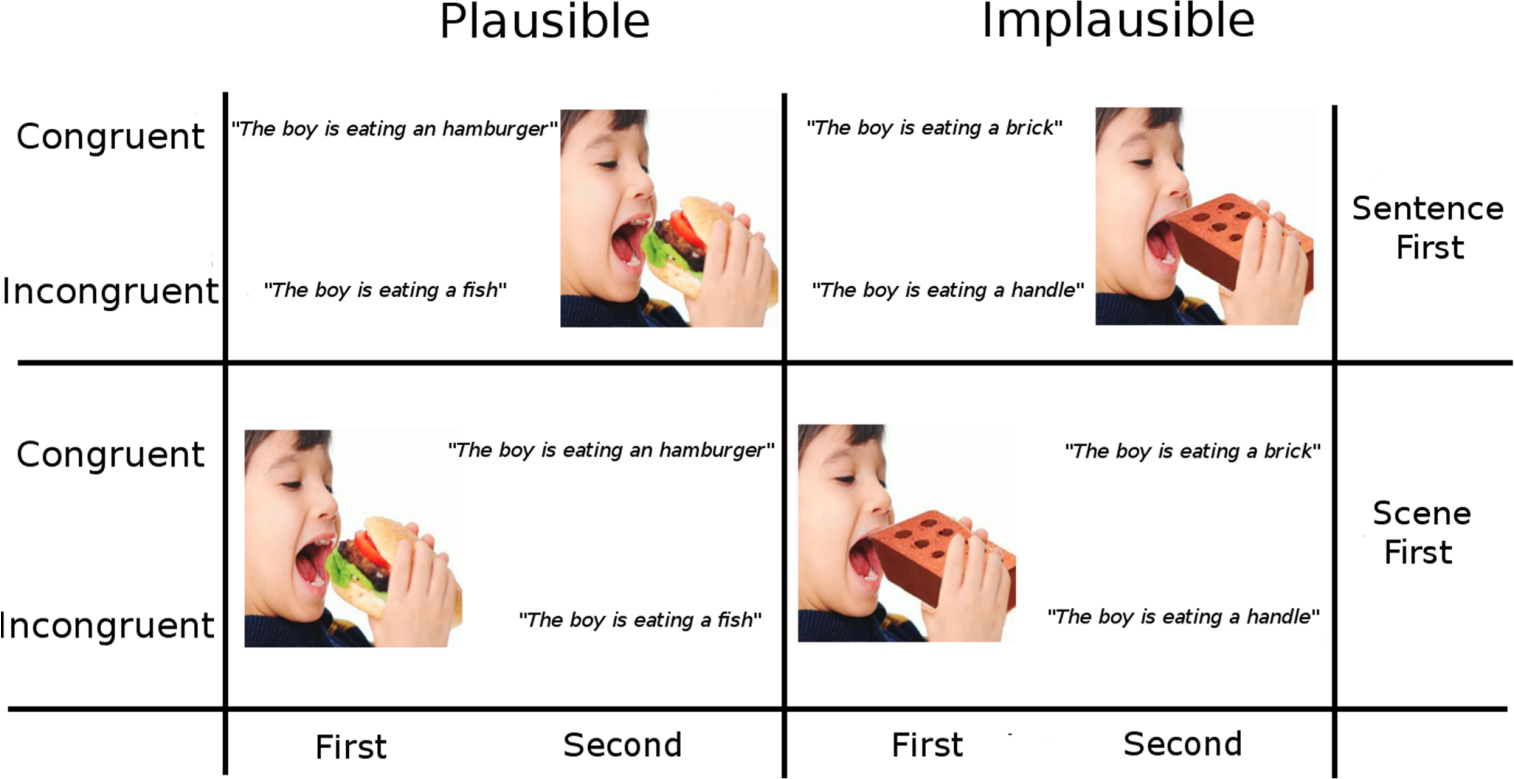
Experimental design and example of experimental stimuli for Order of presentation: Sentence-First (top row) and Scene-First (bottom row), crossing Plausibility and Congruency. For each Order, a sentence or a scene is presented either as a first or as a second stimulus. In Sentence-First, a sentence is read self-paced, then a scene is presented for 1 second. In Scene-First, a scene is presented for 1 second, then a sentence is read. After being exposed to the pair of stimuli, participants are asked to use the computer mouse to evaluate whether the messages conveyed by the two stimuli were congruent or not, see also Fig. 2 for an example of a trial run. The sentence in Portuguese is *o rapaz está a comer ...* and the 4 versions created as: (*um hamburger*, Congruent/ Plausible), (*um tijolo*, Congruent/Implausible), (*um peixe*, Incongruent/Plausible), (*uma alça*, Incongruent/Implausible)

The examination of this interaction requires an extension of typical verification tasks along with novel ways to track competition costs. Beginning with the task, participants in our study were presented with two consecutive mixed modality stimuli (a sentence and a scene) and asked to verify the congruency of the content. As an extension, the content of the stimuli were manipulated to be consistent or inconsistent with prior knowledge expectations (i.e., stimuli convey plausible or implausible content). For example, participants might be asked to read a sentence that is plausible or not (e.g., *they boy is eating a* **hamburger** *vs. eating a* **brick**), and then are shown a visual scene that does or does not match the earlier content (this order is also reversed for another set of participants in a “scene-first” version; see Fig. 1 for the example design). In this way, expectations generated when processing the first stimulus are allowed to interact with a subsequent second stimulus in terms of both plausibility and mutual congruence (and potentially modulated by modality).

The above experimental setup provides a fully-crossed design of congruency and plausibility expectations, which we employ to explore how systematic mismatches of expectations result in different competition costs. We implemented this design under an action dynamics approach that involved tracking participants’ computer-mouse cursor movements during verification - specifically as participants navigated from the bottom of their screens to the top of their screens where “yes” and “no” options were located in opposite corners. In previous work, the resulting movement trajectories have been shown to reveal moment-by-moment competition between the response options as decisions unfold, typically in trajectories that deviate toward a competitor response en route to a target selection (Spivey & Dale, 2004; Magnuson, 2005; Spivey et al., 2005; Farmer et al., 2007; Dale et al., 2007; Duran et al., 2010; Papesh & Goldinger 2012).

In the current paradigm, for example, we may observe response conflicts of this type when the first stimulus conveys plausible information, activating a “yes” response (i.e., initial movement toward this option on the screen), that must be corrected when a subsequent mismatching stimulus appears (to a “no” response). This result would corroborate previous mouse-tracking research that has found response conflicts to be associated with congruency mismatch in verification tasks (e.g., van Vugt & Cavanagh, 2012). Critically, however, our study makes it possible to examine whether conflicts due to violation of congruency expectations are modulated by the plausibility of the stimuli. In particular, we expect that when an initial stimulus conveys information violating prior knowledge, i.e., it is implausible, a ’no’ response is activated. This response must be corrected to a ’yes’ response if the subsequent stimulus is congruent with the initial stimulus, despite the subsequent stimulus also conveying the same implausible content. This is the case, for example, when the participant reads a sentence such as *the boy is eating a brick*, and then he/she is presented with a scene congruently depicting a boy eating a brick. In particular, this response conflict should emerge very early in the initial angle of the movement towards the incorrect response (to a ’no’ response), and be observed throughout the trajectory as a consistent deviation towards the incorrect choice, or as a higher number of directional changes. Such evidence would help refine the predictive processing account by showing that the matching of congruency expectations and bottom- up sensory information is alone not sufficient for facilitating cognitive processing, but much depends on the plausibility of the information being integrated.

## Method

Previous literature has adopted the notion of congruency to indicate both stimulus implausibility (e.g., Mudrik et al., 2010) and mismatch between consecutive stimuli (e.g., West & Holcomb, 2002; Sitnikova et al., 2008). In our 2 ×2 experimental design, *Congruency* indicates whether stimuli matched in content (Congruent, Incongruent) *between* stimuli, and *Plausibility* indicates whether content was expected or unexpected *within* stimuli (Plausible, Implausible). Moreover, the cross-modal *Order* of presentation (Sentence First, Scene First) was manipulated as two separate experiments (between-participants design). In Fig. 1, we provide a schematic description of the experimental conditions for both studies, and provide a full set of crossed pairs of stimuli.

### Participants

Sixty-four students at the University of Lisbon, all native speakers of Portuguese, participated in the study for course credits. The experiment was granted by the Ethics Committee of the Department of Psychology, in accordance with the University’s Ethics Code of Practice.

### Materials

We used 125 photorealistic scenes, originally published in Mudrik et al. (2010), and added another 100 scenes based on open-access material from the Internet (e.g., Flickr). Each of this 225 unique scenes (size = 550 ×550 px) were presented in the two conditions of Plausibility and Implausibility, which means 450 scenes in total between these two conditions (e.g., the picture of the boy either eating a brick, or a hamburger). In order to generate the Congruency conditions, we constructed 2 types of sentence for each condition, for a total of 900 sentences, which is the total number of items^1^. As exemplified in Fig. 1, top row: (a) Congruent/Plausible is the scene of the boy eating an hamburger paired with the sentence *the boy is eating a hamburger*, Congruent/Implausible is the scene of the boy eating a brick paired with the sentence *the boy is eating a brick*, Incongruent/Plausible is the scene of a boy eating a hamburger and the sentence saying *the boy is eating a fish*, Incongruent/Implausible is the scene of the boy eating a brick, and the sentence saying *the boy is eating an handle*.

The sentences were written in Portuguese and checked for grammaticality by two independent native-speaking annotators. The target word (e.g., *hamburger* vs. *brick*) was always positioned at the end of the sentence. The annotators also ensured that the target object depicted in the scene was recognized as the target word used in the sentence.

We divided these 900 stimuli into 4 Latin-Squared lists, 225 items each, such that no items were repeated within the list. From each list, we selected 100 items to present to an individual participant, and made sure that across participants all 900 stimuli, distributed in the 4 lists, were presented an equal number of times.

In order to assess how plausibility, congruency, and other possible task co-variates (such as the grammaticality of the sentence) were perceived during the experiment across trials, we asked the participant at the end of each trial to rate on a scale from 1 to 6 (i.e., from *very strongly disagree* to *very strongly agree*) the: 1) plausibility of the scene, 2) visual saliency of the target object, 3) congruency between the scene and the sentence, 4) grammaticality of the sentence. For these ratings, the previously shown stimuli were displayed and there was no time limit to answer.

In the Supplementary Material, we present analyses of the ratings, in terms of both accuracy and response time, that confirm the validity of our experimental manipulations (’Question answer confidence scores’).

### Apparatus and procedure

The experiment was designed using Adobe Flash 13.0 (sampling at 60 Hz) and run in the computer laboratory of the Department of Psychology at the University of Lisbon. The stimuli were presented on a 21” plasma screen at a resolution of 1024×768 pixels. Participants sat between 60 and 70 cm from the computer screen. Calibration of the mouse position was ensured by forcing participant to click on a black target circle (36 pixels across) located precisely at the bottom-center of the screen at the start of the trial and throughout its different phases. The optical computer mouse was located directly on the table, rather than on a mouse-pad, and participants had enough space around themselves to produce natural responses.

**Fig. 2:**
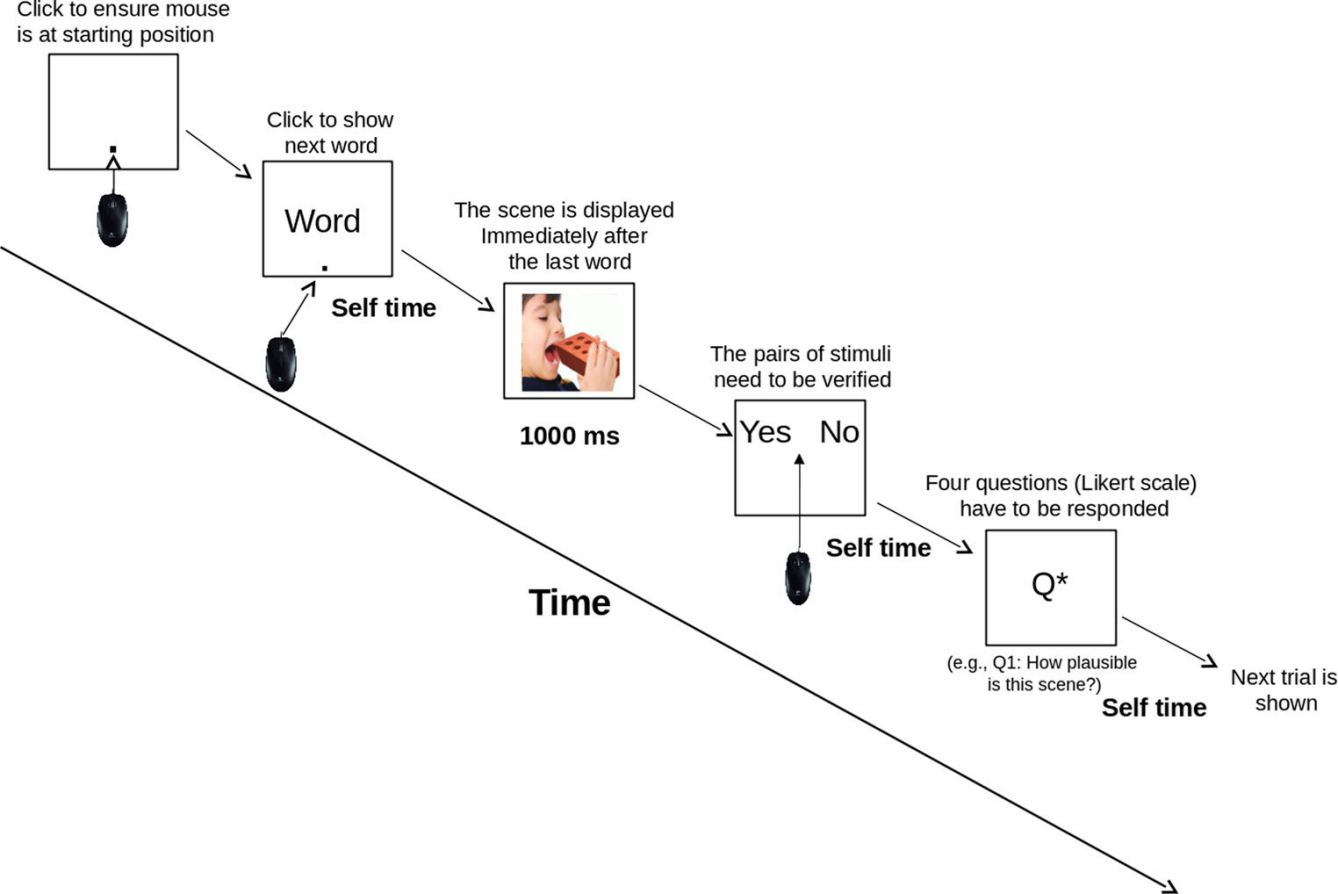
An example of a trial run. A target circle is shown at the beginning of every trial. The target ensures that all participants are calibrated to the same starting position. The target is then clicked to display the sentence one word at time. When the last word is reached, this triggers the presentation of the scene that is displayed for 1000ms. After the display, the yes/no verification buttons, equally spaced from the center of the screen, are shown at the top of the screen. After the decision is made, the participant is asked to rate the four questions on a Likert-scale that gauges: 1) the plausibility of the scene, 2) the visual saliency of the target object, 3) the congruency between the scene and the sentence, 4) the grammaticality of the sentence. For all ratings, the previously viewed scene and sentence are visible, which removes the need to recall the stimuli from memory

Participants first read a sentence, using a word-by-word self-presentation method, by clicking on the calibration button located at the bottom of the screen. After the last word was read, a visual scene was displayed for 1 second. This length of preview time is based on previous work using the same stimuli as Mudrik et al. (2010), and gives enough time to extract scene information and identify the critical target object. The scene then disappeared, and the response options (yes, no) were displayed at the top of the screen, counterbalanced (left/right) between participants. Once the participant clicked on a response, the four questions were presented one at a time across separate screens, after which a new trial started (Fig. 2 illustrates an example trial). The Order condition was simply created by presenting the scene for 1 second prior to the reading of the sentence.

At the beginning of the session, participants performed three practice trials to familiarize themselves with the task; and each completed 100 randomized trials, and spent between 45 and 60 minutes to complete the task.

### Analysis

The dataset we analyzed contained a total of 5,851 unique trials. We removed 8 % (549 trials) from the full dataset if verification times were greater than 4 standard deviations from the mean or due to machine error.

We first analyzed the accuracy of performance during the verification task. This resulted in 5085 accurate trials (≈87 *%*). Based on this set, we analyzed the time to make a decision and the dynamics of the decision process itself (i.e., associated mouse trajectory). In particular, from the verification trajectory, we extracted the following measures: (a) *initial degree* (the degree of deviation from vertical after the mouse trajectory leaves a 50-pixel radius from the starting point, same as Buetti and Kerzel (2009)), where positive values indicate angles towards the incorrect target (b) *latency* (the time taken to move outside an initial region of 50 pixels around the starting point), (c) *x-flips in motion* (the number of directional changes on the x-axis), and (d) the *area under the curve* (AUC) (the trapezoidal area between the trajectory and an imaginary line drawn directly from the calibration button to the correct response bottom). Each measure captures complementary information about the decision process. Initial degree captures the earliest response where conflict can be observed. Latency gets at the initial hesitancy to commit to a decision, whereas x-flips gauges uncertainty and changes of mind as the decision unfolds. Finally, AUC is a summary measure for the overall strength of competition toward an incorrect response, where greater area suggests stronger competition. These measures have been detailed in previously published research (e.g., Buetti and Kerzel, 2009; Freeman & Ambady, 2010; Dale & Duran, 2011).

To perform our analysis, we employed linear mixed-effects models based on the R statistical package lme4 (Bates et al. 2011). We built full models with all main effects and possible interactions with a maximal-random structure, where each random variable of the design (e.g., Participants), is introduced as intercept and as uncorrelated slope on the predictors of interest (e.g., Plausibility), see Barr et al. (2013). We adopted this approach to also account for the large variance observed across participants in mouse-tracking experiments (Bruhn et al. 2014).

The dependent measures examined are: Accuracy^2^ (probability), Response Time (in seconds), Initial Degree (in degrees), Latency (in seconds), x-flips (count), and AUC. The fixed variables of our design are Congruency (Congruent, Incongruent), Plausibility (Plausible, Implausible) and Order (Scene-First, Sentence-First). The random variables of our design are Participants (64), Scenes (450, as we have 225 scenes in two conditions of Plausibility) and Counterbalancing (2, left and right). We report tables with the coefficients of the predictors, their *t*-values, and indicate their *p*-value significance.

## Results

We start by examining the performance accuracy and response time across all experimental conditions. We then examine how the response conflict develops along the decision trajectory by looking at the measures of initial degree, latency, x-flips, and area under the curve, which characterize its underlying dynamics. We report both the raw observed data (mean and SD), as well as the coefficients of the mixed-effects maximal model.

### Accuracy and reaction time

When looking at the response accuracy, we find it to be quite high overall (≈87 *%*), indicating that participants are able to perform the verification task correctly (refer to Table 1). Accuracy is strongly mediated by Plausibility and Congruency, as well as by the Order used to present the sentence/scene pairs. In particular, participants are more accurate when plausible stimuli are presented, and when the sentence is presented prior to the scene (refer to Table 2 for the model coefficients and their significance). Congruency is not significant as a main effect, but only in interaction with Plausibility, where implausible and congruently matching stimuli are verified less accurately, independent from the Order of presentation.

**Table 1:** Observed data (mean and standard deviation) of all summary measures reported in the study, which are organized in the table by order of presentation (Sentence-First, Scene-First), Congruency (Congruent, Incongruent) and Plausibility (Plausible, Implausible)

	Sentence-First				Scene-First			
	Congruent		Incongruent		Congruent		Incongruent	
	Plausible	Implausible	Plausible	Implausible	Plausible	Implausible	Plausible	Implausible
Accuracy (*%*)	0.92 ±0.27	0.87±0.34	0.88±0.32	0.88±0.33	0.88±0.32	0.78±0.40	0.85±0.33	0.87±0.35
Response time (second)	1.4 ±0.56	1.52±0.57	1.41±0.56	1.43±0.57	1.36±0.46	1.45±0.53	1.41±0.50	1.41±0.50
Initial degree (degree)	−2.37 ±50.48	−1.13±48.33	−4.22±52.79	−5.34±48.39	−5.62±37.58	−9.01±45.51	−5.71±38.95	−6.26±42.58
Latency (second)	0.46 ±0.28	0.50±0.31	0.45±0.28	0.48±0.31	0.46±0.21	0.47±0.24	0.49±0.25	0.49±0.25
X-flips (count)	1.40 ±1.14	1.50±1.30	1.40±1.15	1.35±1.1	1.38±1.07	1.47±1.26	1.36±1.14	1.28±1.13
Area under curve (AUC)	0.72 ±0.4	0.77±0.41	0.69±0.37	0.7±0.4	0.69±0.39	0.71±0.4	0.7±0.41	0.7±0.4

**Table 2:** Coefficients of mixed-effects models with maximal random structure (intercept and slopes on Participants, Scenes and Counterbalancing). Each dependent measure, organised across columns, is modelled as a function of the centred and contrast coded predictors: *Congruency* (Congruent = 0.5, Incongruent = -0.5), *Plausibility* (Plausible = 0.5, Implausible = -0.5), *Order* (Sentence-First = -0.5, Scene-First = 0.5). We report the *β* with the associated p-value, and the t-value from which it was derived

	Accuracy		Response Time		Initial-Degree		Latency		X-Flip		AUC	
Dependent measures	*β*	t	*β*	t	*β*	t	*β*	t	*β*	t	*β*	t
Intercept	2.87 ^∗∗∗^	23.97	1.4 ^∗∗∗^	53.67	−4.76 ^∗∗∗^	−3.7	0.47 ^∗∗∗^	33.86	0.29 ^∗∗∗^	9.76	0.71 ^∗∗∗^	55.42
Plausibility	0.51 ^∗∗^	3.31	−0.07 ^∗∗∗^	−3.6	−0.02	−0.01	−0.02 ^∗^	−2.46	−0.02	−0.71	−0.01	−1.34
Congruency	0.005	0.04	0.02	1.11	1.39	0.45	−0.007	−0.77	0.07	1.72	0.03	0.91
Order	−0.45 ^∗∗^	−3.17	−0.02	−0.5	−3.15	−1.22	0.01	0.35	−0.05	−0.76	−0.02	−0.84
Plausibility: Congruency	0.83 ^∗∗^	2.7	−0.06 ^∗^	−2.1	−5.64 ^∗^	−2.24	−0.001	−0.52	−0.17 ^∗∗∗^	−3.69	−0.04 ^∗∗^	−2.02
Plausibility: Order	0.26	1.25	0.04	1.43	−.11	−0.44	0.04 ^∗^	2.46	0.03	0.75	0.02	1.25
Congruency: Order	−0.49	−1.51	−0.06	−1.51	−4.92	−0.79	−0.03 ^∗^	−2.14	0.06	0.78	−0.06	−0.96
Plausibility: Congruency: Order	0	0.01	−0.01	−0.19	0.19	0.04	0.001	0.05	−0.1	−1.12	0.04	1.04

* *p*<0.05, ** *p*<0.01, *** *p*<0.001

On the response times to take a decision (correct trials only), we observe again a main effect of Plausibility, whereby implausible information is verified slower than plausible information. Moreover, we confirm the interaction between plausibility and congruency, whereby implausible but congruent stimuli take longer to be verified.

### Initial degree, latency, x-flip, and area under the curve

Table 2 reports the coefficients for the mixed-model analyses of the initial degree, latency to start the movement, the flips along the x-axis during the trajectory, and the area sub-tending it.

From the very first moments of the trajectory (initial degree), the participants display a larger conflict in their response when the stimuli are implausible and congruently matched (two-way interaction with Plausibility and Congruency). This can be also seen from the observed data in Table 1 where we observe a more positive initial angle - with positive indexing an angular movement towards the incorrect response - when implausible stimuli are congruent. Conversely, the least deviated initial angle is observed when implausible stimuli are incongruent. Nothing else is observed as main effects, nor as interactions, with initial angle.

For latency, participants hesitate more before starting their verification when the stimuli are implausible, a pattern similar to that observed with overall response time. Interesting results on the latency are obtained as interactions with Order of presentation. We find that when the scene is presented first and the stimuli are implausible, the participants are faster to initiate the movement. We also find a significant interaction between Congruency and Order, such that when the scene is presented first, and the stimuli are incongruent, participants take longer to initiate the movement.

The measures of x-flip and area under the curve converge on the same significant result: implausible but congruent pairs generate more complex trajectories (interaction with Plausibility and Congruency). These results corroborate all other analyses reported above: greater conflicts on the response are generated when implausible stimuli have to be accepted as congruent.

In the Appendix, we also model the angular trajectories using growth-curve analysis, corroborating the results reported above at an even finer-grained resolution. Furthermore, in the Supplementary Material, we re-analyze all summary measures presented in this study, but instead of using the two-level categorical distinction for Plausibility (Plausible and Implausible) and Congruency (Congruent and Incongruent) as predictors, we use the ratings provided by the participants during question answering (refer to Fig. 2 for an example of experimental trial with these ratings). This additional analysis largely confirms the results presented in the main paper, and serves to demonstrate the ecological validity of our experimental manipulations and verification paradigm.

## Discussion

There has been a recent and growing interest in understanding the cognitive system along central principles of predictive processing (e.g., Clark, 2013; Pickering & Clark, 2014; Lupyan & Clark, 2015). The current study adds to these attempts by employing a task that required participants to verify whether two sequentially presented stimuli, which also varied in plausibility of content, conveyed the same message. In doing so, two sources of expectancy (stimuli congruency and stimuli plausibility) were allowed to converge and compete during verification. Moreover, in a departure from the majority of previous research, we examined this competition in the continuous updating of the motor system using an action dynamics approach. This allowed a novel view into the temporally-extended activation and resolution of competition. Whereas reaction time primarily informs the time it takes to make a response, and EEG on the neural resources that are recruited at a specific time, we were able to examine competition across the earliest moments of processing and throughout a decision process.

Our analysis of the motor movements focused on key summary measures, including initial degree of movement, latency of movement, x-flips, and area under the curve (AUC). We interpreted greater latencies, increased complexity, and deviations toward an incorrect response as signaling greater processing costs and the application of error-signal updating. All measures consistently point to the same core result: implausible but congruently matching stimuli generated greater response competition than incongruent and implausible stimuli. A match between expectations elicited by an initial stimulus and incoming sensory information from the second stimulus would normally facilitate processing. However, in our scenario, such facilitation was actually disrupted because the incoming information violated other expectations based on prior knowledge.

As evidenced by the computer mouse movements, when the implausible stimulus is encountered, there is a very early hesitation and bias to respond “no” that is activated and persists during verification, competing with the congruency expectation to respond “yes.”

This result is in line with current proposals of predictive processing by underscoring how the cognitive system is actively engaging generative mechanisms of active expectation (i.e., predictive coding) and error correction (e.g., Rao & Ballard, 1999; Bar, 2007; Hinton, 2007; Kok et al., 2012; Wacongne et al., 2012), drawing on multiple sources of expectations from the local context (congruency expectations) and from prior knowledge (plausibility expectations). Moreover, this research helps refine current proposals of predictive processing by showing that congruency expectations can be immediately disrupted and temporarily overridden when incoming stimuli is in violation of prior knowledge.

The cross-modal paradigm also tested whether the directionality of the response costs are sensitive to the modality order of presentation (i.e., sentence-first vs. scene-first) (e.g., Clark & Chase, 1972; Federmeier & Kutas, 2001; Pecher et al., 2003). We found hints of an asymmetry on the latency to start the movement, especially in the angular trajectory (see Appendix), whereby stronger conflicts are observed when an implausible sentence is reinforced by a subsequent congruently matching scene (sentence-first scenario). Based on the literature on simulation models in sentence-picture verification tasks (e.g., Zwaan et al., 2002; Ferguson et al., 2013), we speculate that when a sentence is presented first, the resulting simulated imagery, opposed to merely seeing a picture, relies on deeper connections to prior knowledge to generate its content, and thus such condition shows a greater sensitivity to stimulus plausibility. Future research will be needed to better assess these asymmetries triggered by modality order by providing, for example, greater initial linguistic context to make the simulations even richer.

Another natural avenue of research for investigating plausibility and congruency on predictive processing is to use a fully crossed design, as in the current study, with EEG measures. Intriguing results about the impact of such sources on neural responses have been reported, for example, by Dikker and Pylkkanen (2011), who used a picture-name matching task and observed a very early signature of prediction violation, at around 100ms, when words did not accurately describe the pictures. This result has recently been corroborated by Coco et al. (2015), where congruency and plausibility were found to be associated with distinct temporal latencies, such that incongruent pairs generated revision costs as early as 100ms after the stimulus onset, whereas implausibility begins at 250ms (e.g., same latency of Mudrik et al. (2010) and more recently (Mudrik et al. 2014)). Of great interest would be to compare neural responses with the behavioral patterns reported here and draw joint conclusions about on-line stimuli processing and later integrative verification dynamics. Other key questions relate to the adaptation (or not) of participants to implausible verification, and the amount of exposure needed to modify prior knowledge so that implausible information is accepted without additional costs.

In conclusion, the current study provides new evidence for how response conflicts arise in the motor system as decision responses are acted out. We take this as demonstrating that the plausibility of stimuli mediates congruency expectations in a verification task. More generally, these results provide compelling evidence in support of a predictive processing account by showing that the matching of congruency expectations is not sufficient for facilitating cognitive processing, but depends greatly on the plausibility of information that is being integrated.

## Figures and Tables

**Fig. 3:**
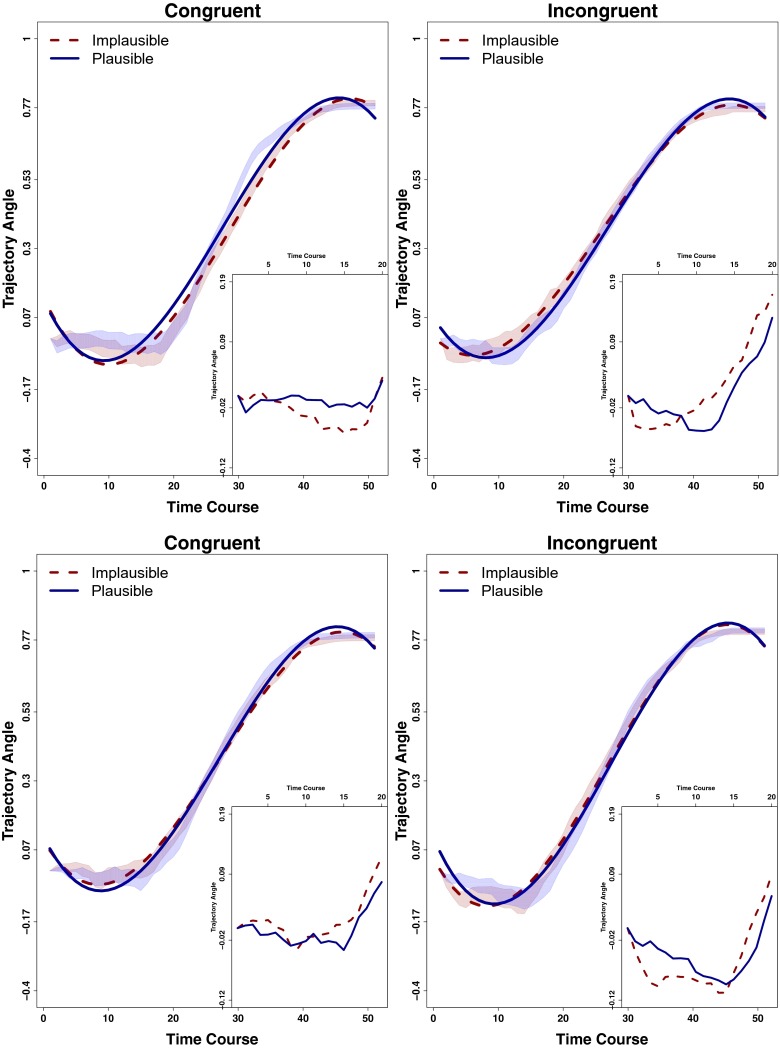
Angular trajectory over 51 interpolated time-bins for the two Order of Presentation: Sentence-First (top-row); Scene-First (bottom-row). Congruency is organized as panels (Congruent- Left; Incongruent - Right), while Plausibility is plotted within each panel as line types (Plausible - solid line; Implausible - dotted line). The shaded bands indicate the standard error around the observed mean. The lines represent the predicted values of the LME model reported in Table 3. In the right corner of each plot, we zoom into the first 20 bins of the observed trajectory to highlight its earliest revision-dynamics

## Appendix: Angular verification trajectories

In this Appendix, we report a finer-grained analysis of the angular trajectory, which largely corroborates the results presented in the main text; and provides greater detail about the role played by Order of presentation.

We visualize the x,y trajectory over time as angular values along the x-axis, which is where the most obvious changes in the trajectory happen (Scherbaum et al. 2010; Dshemuchadse et al. 2013). The angle is calculated as the tangent along the trajectory with values above 0 conventionally representing the direction towards the correct response option. Negative angular values would instead be regarded as movement toward the incorrect, competitor response option. As trials were self-terminated, the trajectories differed in their number of time points. We adopted the approach proposed by Spivey et al. (2005) where trajectories are linearly interpolated (up- or down-sampled) to be scaled all within 51 time-bins, which is around the mean (56.35±30.64) and median (51) number of time-points observed overall.

Angular trajectory is a dependent measure that unfolds over the normalized time bins. Thus, in our linear-mixed effects model, we add a predictor for Time, which we represent as an orthogonal polynomial of order three and captures the curvilinear dynamic of the trajectory (see Mirman (2014) for a detailed explanation of the framework). The linear term of the polynomial has exactly the same interpretation as a linear regression of the dependent measure over time (Time ^1^). In the trajectory, this term points at the overall change in the direction. The quadratic term can be used to identify sudden changes in the linear trend, e.g., a decrease followed by an increase (Time ^2^). In the trajectory, this term points at the overall shape of the direction, i.e., whether it tends to go towards the correct or competitor response. The cubic term can be used to identify early and late effects in the trend (Time ^3^). In the trajectory, this term points at whether it initially deviates towards the competitor, i.e., negative values, or the correct response.

We visualize observed data as shaded bands indicating the standard error around the mean, and overlay the mean estimate of model fits as lines. We report tables with the coefficients of the predictors, significant at *p*<.05, their standard error, and derive *p*-values from the *t*-values for each of the factors in the model. The *t*-distribution converges to a *z*-distribution when there are enough observations (our data contains 264,180 points), and hence we can use a normal approximation to calculate *p*-values.

In Fig. 3, we plot the angular trajectory followed by the participants as they correctly verified the Congruency of the pair of stimuli (Sentence-First, top-row; Scene-First, bottom-row); and zoomed in on the earliest section of the trajectory to visualize the initial stages of the decision process. In order to simplify the understanding of our results, we focus on the significant terms, and especially on those corroborating with the summary measures reported above in the main text (refer to Table 3 for model coefficients).

**Table 3:** Mixed-effect maximal model analysis of the angular trajectory

Predictor	*β*	*SE*	*t*	*p*
Intercept	0.344	0.069	4.973	.00001
Time ^1^	2.287	0.044	51.217	.00001
Time ^2^	0.187	0.057	3.25	.001
Time ^3^	−0.602	0.035	−16.964	.00001
Congruency:Time ^1^	−0.057	0.015	−3.621	.001
Congruency:Time ^2^	0.143	0.015	9.004	.00001
Congruency:Time ^3^	−0.031	0.015	−1.995	0.05
Plausibility:Time ^3^	−0.076	0.035	−2.136	0.03
Congruency:Plausibility:Time ^2^	−0.164	0.031	−5.191	.00001
Congruency:Plausibility:Order	0.034	0.009	3.545	0.001
Plausibility:Order:Time ^2^	−0.080	0.030	−2.629	0.01
Congruency:Order:Time ^1^	0.178	0.031	5.746	.00001
Congruency:Order:Time ^2^	0.232	0.031	7.509	.00001
Congruency:Order:Time ^3^	−0.170	0.031	−5.52	.00001
Congruency:Plausibility:Order:Time ^1^	−0.156	0.062	−2.519	0.01
Congruency:Plausibility:Order:Time ^2^	−0.154	0.062	−2.493	0.01

The fixed effects of the model, contrast coded, are: *Congruency* (Incongruent: −0.5, Congruent: 0.5), *Plausibility* (Implausible: −0.5, Plausibile: 0.5), Order (Scene-First = -0.5, Sentence-First = 0.5). Time (51 bins) is represented as an orthogonal polynomial of order three (Time ^1^, Time ^2^, Time ^3^). Random intercepts and slopes on Participant (64) and Scene (450)

Almost all effects on the trajectory are captured by interactions between Congruency, Plausibility, and Order, with the polynomial terms of Time. We find that incongruent verifications are re-directed more decisively towards the correct response (Congruency:Time ^1^), especially later in the trajectory (Congruency:Time ^3^), but have an overall negative bowing, i.e., more deviated pull towards the incorrect response than congruent trials (Congruency:Time ^2^). We also find implausible trials displaying an early deviation towards the incorrect response (Plausibility:Time ^3^).

When looking at three-ways interactions, we find that for implausible, congruently matched stimuli, these generate a more protracted deviation in the trajectory than incongruent trials (Congruency:Plausibility:Time ^2^). This result corroborates the summary measures reported in the main text. Moreover, this pattern is particularly prominent when the sentence is presented first (refer to the *β* for the three-way interaction Congruency:Plausibility:Order in Table 3) This effect is also rather evident in Fig. 3, top -row (Sentence-First), where there is consistent deviation towards the incorrect response, lasting until the end of the trajectory.

Further examination of Order reveals that when the scene is presented first, incongruent verifications deviate more strongly in the initial phases of the trajectory (Congruency:Order:Time ^3^); such deviations are quickly recovered as indicated by the positive three-ways interaction of Congruency:Order:Time ^1^ and Congruency:Order:Time ^2^. For Plausibility instead, when the scene is presented as a second stimulus, we find the trajectory to present an overall negative bowing toward the incorrect response (Plausibility:Order:Time ^2^).

Finally, when looking at the four-way interaction, we find that after an initial hesitation, participants corrected their arm movement trajectories towards the correct response more rapidly when implausible stimuli congruently matched, and the sentence served as the initial stimulus, i.e., a positively linear trajectory with an upward bowing trend.

In summary, on arm movement trajectories, we confirm electrophysiology research, whereby implausible stimuli are associated with greater competition costs. Also, incongruency causes an overall more deviated trajectory. However, in the current paradigm examining response movement dynamics, effects occur also after the stimuli had been initially encountered. In addition, our study also shows that implausible but congruently matching stimuli generate higher competition costs than incongruent and implausible stimuli. In this case, implausible messages have to be accepted as congruent, even though they violate prior knowledge, i.e., boys do not normally eat bricks. This result was particularly strong when the sentence was presented prior to the scene. The effects of Order of presentation were captured in the angular trajectory but not in the summary measures, because here we include the time-course component, and have a much larger number of data points to estimate the parameter coefficients.

## Footnotes

1 Note that the incongruent condition could also have been obtained by fixing the plausibility of the sentence (plausible or implausible) and manipulating the associated scenes (four versions). However, as scene material is harder to construct, we opted against this method.

2 Note: for the dependent measures of accuracy and x-flips, we utilized the glmer function with family set as binomial and poisson respectively, in order to assign the linking function appropriate to the type of data. For this reason, all estimates of accuracy should be transformed using the probit transformation, if probabilities have to be reconstructed.
